# Risk Prediction of Metachronous Colorectal Cancer from Molecular Features of Adenomas: A Nested Case–Control Study

**DOI:** 10.1158/2767-9764.CRC-23-0186

**Published:** 2023-11-13

**Authors:** Henriette C. Jodal, Eddymurphy U. Akwiwu, Margriet Lemmens, Pien M. Delis-van Diemen, Dagmar Klotz, Leticia G. Leon, Soufyan Lakbir, Meike de Wit, Remond J.A. Fijneman, Monique E. van Leerdam, Evelien Dekker, Manon C.W. Spaander, Gerrit A. Meijer, Magnus Løberg, Veerle M.H. Coupé, Mette Kalager, Beatriz Carvalho

**Affiliations:** 1 Clinical Effectiveness Research Group, University of Oslo, Oslo, Norway.; 2 Clinical Effectiveness Research Group, Oslo University Hospital, Oslo, Norway.; 3 Section of Oncology, Drammen Hospital, Vestre Viken Hospital Trust, Drammen, Norway.; 4 Department of Epidemiology and Data Science, Amsterdam Public Health Research Group, Amsterdam University Medical Centers, Location VU Medical Center, Amsterdam, the Netherlands.; 5 Department of Pathology, The Netherlands Cancer Institute, Amsterdam, the Netherlands.; 6 Department of Pathology, Oslo University Hospital, Oslo, Norway.; 7 Bioinformatics Group, Department of Computer Science, Vrije Universiteit Amsterdam, Amsterdam, the Netherlands.; 8 Department of Gastrointestinal Oncology, Netherlands Cancer Institute, Amsterdam, the Netherlands.; 9 Department of Gastroenterology and Hepatology, Leiden University Medical Center, Leiden, the Netherlands.; 10 Department of Gastroenterology and Hepatology, Amsterdam University Medical Centers, Location Academic Medical Center, Amsterdam, the Netherlands.; 11 Department of Gastroenterology and Hepatology, Erasmus MC University Medical Center, Rotterdam, the Netherlands.

## Abstract

Current morphologic features defining advanced adenomas (size ≥10 mm, high-grade dysplasia or ≥25% villous component) cannot optimally distinguish individuals at high risk or low risk of metachronous colorectal cancer (me-CRC), which may result in suboptimal surveillance. Certain DNA copy-number alterations (CNAs) are associated with adenoma-to-carcinoma progression. We aimed to evaluate whether these molecular features can better predict an individual's risk of me-CRC than the morphologic advanced adenoma features.

In this nested case–control study, 529 individuals with a single adenoma at first colonoscopy were selected from a Norwegian adenoma cohort. DNA copy-number profiles were determined, by low-coverage whole-genome sequencing. Prevalence of CNAs in advanced and non-advanced adenomas and its association (OR) with me-CRC was assessed. For the latter, cases (with me-CRC) were matched to controls (without me-CRC) on follow-up, age and sex.

CNAs associated with adenoma-to-carcinoma progression were observed in 85/267 (32%) of advanced adenomas and in 27/262 (10%) of non-advanced adenomas. me-CRC was statistically significantly associated, also after adjustment for other variables, with age at baseline [OR, 1.14; 95% confidence interval CI), 1.03–1.26; *P* = 0.012], advanced adenomas (OR, 2.46; 95% CI, 1.50–4.01; *P* < 0.001) and with the presence of ≥3 DNA copy-number losses (OR, 1.90; 95% CI. 1.02–3.54; *P* = 0.043).

Molecularly-defined high-risk adenomas were associated with me-CRC, but the association of advanced adenoma with me-CRC was stronger.

**Significance:**

Identifying new biomarkers may improve prediction of me-CRC for individuals with adenomas and optimize surveillance intervals to reduce risk of colorectal cancer and reduce oversurveillance of patients with low risk of colorectal cancer. Use of DNA CNAs alone does not improve prediction of me-CRC. Further research to improve risk classification is required.

## Introduction

Colorectal cancer develops from benign precursor lesions, known as adenomas and serrated polyps, through a series of genetic and epigenetic changes that can take 10 to 20 years (1–3). Colorectal adenoma incidence rises with age, and one or more adenomas are found in about 30% of men and 20% of women over 50 years of age at screening colonoscopy (4, 5). However, given a lifetime colorectal cancer risk of around 5%, the majority of adenomas will never progress to cancer (6). Sessile serrated lesions are substantially less common than adenomas and knowledge on their progression to cancer is less well understood (7, 8). Individuals who have had adenomas removed are classified as being at high or low risk of metachronous colorectal cancer (me-CRC), dependent on the size, number, and histopathologic features of the removed polyps. Individuals have been considered to be at high risk when diagnosed with an advanced adenoma (AA; size ≥10 mm, high-grade dysplasia or ≥25% villous component) or ≥3 adenomas, and at low risk when they had one to two tubular adenomas <10 mm of size, with low-grade dysplasia (9, 10). In 2020, the European Society of Gastrointestinal Endoscopy (ESGE) changed their definition of individuals at high risk in light of new evidence, to only include individuals with at least one adenoma (or serrated polyp) with size ≥10 mm or high-grade dysplasia, or with five or more adenomas regardless of histology (11).

In a population-based study, we found that individuals who had AAs removed had only slightly higher colorectal cancer mortality than the general population (12). This suggests that detection and removal of AAs is not a very precise predictor of the risk of developing and dying from colorectal cancer. The current recommendations for post-polypectomy surveillance thus may cause large numbers of unnecessary colonoscopies. The latter is of importance giving both the burden of colonoscopy and the suboptimal cost-effectiveness of current surveillance recommendations (13, 14). Better predictors of me-CRC risk are therefore needed.

Chromosomal instability (CIN) is the major pathway involved in adenoma-to-carcinoma progression (15). We have previously demonstrated that in adenomas harboring a focus of cancer, the phenotypically benign part of the lesion contained most of the chromosomal alterations that were also present in the cancer focus, indicating a role of these in adenoma-to-carcinoma progression, therefore named as cancer-associated events (CAE; refs. 16, 17). The presence of two or more of these CAEs had an accuracy of 80% to discriminate progressed from non-progressed adenomas (16). We further showed, in a longitudinal study, where adenomas were left *in situ* and re-evaluated and resected 3 years later, that CAEs were present in adenomas with higher growth rates and absent in adenomas that regressed (18). In a previous pilot study, we found that only 23%–36% of AAs and 2%–5% of non-AAs showed presence of ≥2 CAEs (19).

Moreover, in studies that modeled colorectal adenoma-to-carcinoma progression by perturbing organoids with gene mutations (20, 21), only organoids derived from adenomas, which during culture acquired DNA copy-number alterations (CNAs) next to the introduced mutations, were able to form invasive tumors in mice, confirming the importance of these CNAs for the cancer phenotype (20).

At the level of an individual adenoma, we now know that CIN, resulting in specific gross chromosomal changes, is strongly associated with progression of that adenoma to cancer. We hypothesize that when an individual carries an adenoma already showing the molecular alterations highly associated with progression to cancer (e.g., CAEs), such an individual may be prone to later develop another adenoma that also accumulates these CAEs and does progress into a me-CRC. Therefore, using molecular features to predict the risk of adenomas to progress to cancer could lead to more precise identification of individuals at risk of me-CRC.

In this study, we investigated whether a molecularly-defined high-risk adenoma, that is, ≥2 DNA CAEs, is a better predictor for me-CRC than the currently used morphologic features defining AA. Next to CAEs, we also considered three other definitions of molecular high-risk, that is, the presence of ≥3 CNA (gains and/or losses), the presence of ≥3 copy-number gains, and ≥3 copy-number losses. We utilized a previously established large adenoma cohort, with well-documented long-term outcome of me-CRC (12), to determine the prevalence of DNA CNAs in advanced and non-AAs. By using a matched case–control design nested within this cohort, we investigated the association between me-CRC and presence of an AA or a molecularly-defined high-risk adenoma at baseline.

## Materials and Methods

### Description of the Adenoma Cohort

**FIGURE 1:**
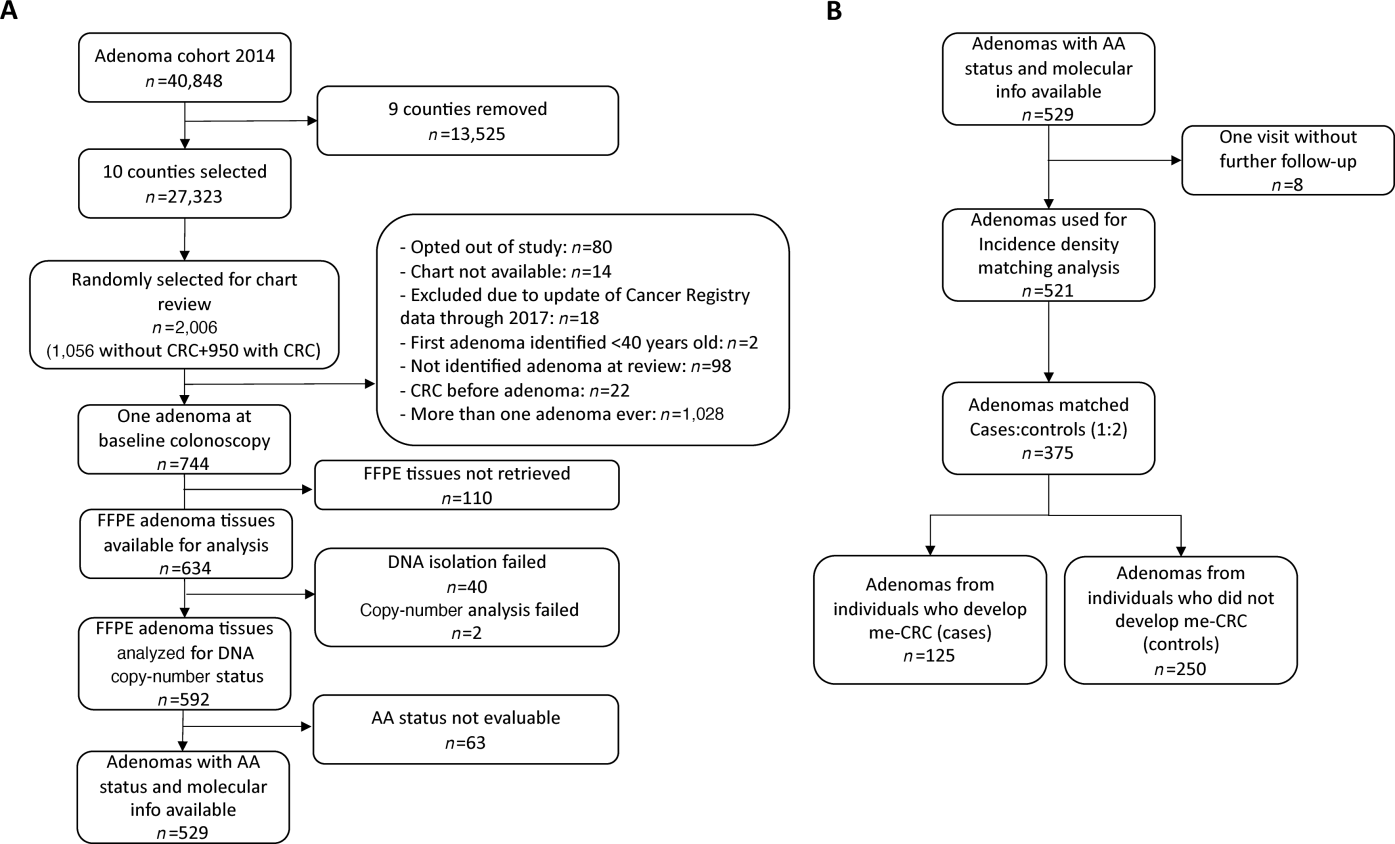
Flowchart of the selection and analysis of the study cases. **A,** Selection of samples with complete molecular and morphologic information available. **B,** Selection of samples used in the case–control comparison (incidence density matching analysis). FFPE, formalin-fixed paraffin-embedded; AA, advanced adenoma; me-CRC, metachronous colorectal cancer.

The design of the adenoma cohort is described elsewhere (12, 22). In brief, the cohort consists of all individuals aged 40 years or older, mostly with symptoms, living in Norway who had adenomas removed between January 1, 1993, and December 31, 2007, a total of 40,848 individuals (Fig. 1A). The individuals were identified from the Cancer Registry of Norway, where they were registered with topographical ICD-O-3 codes 180, 182 through 189, 199, or 209, combined with morphologic ICD-O-3 codes 8140, 8210, 8211, 8261, or 8263 (i.e., adenomas). Individuals with familial adenomatous polyposis were identified through linkage with the Norwegian Polyposis Registry and excluded from the cohort. Individuals were followed through linkage to the Cancer Registry and the Norwegian Cause of Death Registry until December 31, 2018. Median follow-up time was 13.0 years, interquartile range (IQR), 7.3–17.0 years.

### Sample Size Calculation

For this study, two sample size calculations were done. First, for the estimation of the prevalence of high-risk molecular features in individuals with AA and with non-AA, respectively, we aimed for an accuracy of 2.5% SE and 1.25% (SE) around the expected proportions of 25% and 3%, based on our previous work (19). Using the standard formula for the standard error of a proportion, the required sample size of AA and non-AA was 300 and 187, respectively.

Second, we aimed to be able to detect an at least 3-fold increased colorectal cancer risk (measured by the OR) in individuals carrying adenoma with high-risk molecular features compared with those without high-risk molecular features, under the null hypothesis of absence of such relationship. This OR of 3 was chosen because, to replace AAs as an intermediate endpoint, molecular features should have a substantially stronger association with colorectal cancer than the expected OR for association between AA and colorectal cancer of around 2 (12). We use a nested case–control design with incidence density matching of one case to two controls from the Norwegian surveillance adenoma cohort, that is, controls are selected from those persons who survive without developing colorectal cancer at least as long as corresponding cases. In the full cohort, 48% of individuals had AAs and 52% had non-AAs. Given the above-mentioned expected prevalence of high-risk molecular features in individuals with AA and non-AA of 25% and 3%, respectively, the expected prevalence of high-risk molecular features in the whole cohort is 13.6%. Under the null hypothesis, the proportion of high-risk molecular features in colorectal cancers and controls would simply equal a weighed sum of the expected proportion of these features in AAs and non-AAs. That is, in colorectal cancers (62% AA, 38% non-AA) and controls (46% AA, 54% non-AA), this expected proportion is 16.7% and 13.1%, respectively (12, 19). These expected proportions under the null hypothesis were shifted upward (for colorectal cancer) and downward (for controls) such that the OR for association between molecular high-risk features and colorectal cancer equals 3 while maintaining equal average proportion of high-risk molecular features. Under a 32% prevalence of molecular high-risk features at baseline in colorectal cancers and a 13% prevalence of molecular high-risk features in controls, an alpha of 5% and a power of 90%, a matched case–control study with 69 cases and 137 controls is able to detect a significant 3-fold association between colorectal cancer and high-risk molecular features.

### Selection of Adenomas

We restricted this nested case–control study to individuals living in 10 out of 19 counties in Norway, constituting 79% of the country's population (https://www.ssb.no/en/befolkning), as described previously (23). Of these, 1,005 individuals developed me-CRC, of which a random sample of 950 was selected for chart review. Among the remaining individuals who did not develop me-CRC, 1,056 individuals were randomly chosen for chart review (Fig. 1). All selected individuals who were alive were contacted and given the opportunity to opt out of the study, which 80 individuals did. We excluded individuals whose patient charts were unavailable (*n* = 14), or whose entries in the Cancer Registry were later removed because of correction of the data (*n* = 18). After chart review, 122 individuals were excluded for the following reasons: (i) first adenoma was removed before age 40 years (*n* = 2), (ii) a diagnosis of colorectal cancer before first adenoma removal (*n* = 22), or (iii) inability to identify any adenoma at chart review (*n* = 98). For any individual who had more than one adenoma removed from the same colonic segment in the same year, we assumed that this was the same adenoma. If the histology of these adenomas differed, we selected the most advanced histology for analysis.

For the purpose of this study, we only included individuals who had had one adenoma removed at baseline (*n* = 744) and from whom we could acquire the formalin-fixed paraffin-embedded (FFPE) adenoma tissue from the pathology department at the hospital where the adenoma was resected (*n* = 634; Fig. 1).

### DNA Isolation and Copy-number Profiling

From each FFPE tissue block, 5–10 sections of 10 µm, depending on the tumor area available, were used for DNA extraction. DNA was isolated using the Qiagen Allprep DNA/RNA micro kit (Qiagen Benelux B.V.) as described previously (24). DNA concentration and quality was evaluated with Nanodrop (One Microvolume UV-Vis Spectrophotometer 2022, Thermo Fisher Scientific Inc.), being a ratio 260 nm/280 nm between 1.8 and 2.0, considered high quality. Good-quality double-strand DNA was also evaluated using Qubit (Life Technologies, 2014, Thermo Fisher Scientific Inc.).

DNA CNAs were determined by means of low-coverage whole-genome sequencing (WGS; ref. 25). Briefly, after library preparation, DNA was sequenced on a HiSeq 2500 (Illumina) on a 65 bp single read. Sequences were aligned to reference genome build hg38. Low-coverage (0.2x) WGS copy-number data were processed as described previously (19). Briefly, QDNAseq (25) was used to divide the genome into nonoverlapping fixed-sized bins of 100 kb, and for each sample, estimates of the copy number were determined by counting the number of reads in each bin. After segmentation of the copy-number data, copy-number calls (loss, normal, gain) were done using the Bioconductor/R-package CGHcall (26). Downstream analysis of processed data (called for DNA copy-number status) focused on the CNAs previously described to be associated with adenoma to carcinoma progression (16), the CAEs, namely 8q, 13q, and 20q gains and 8p, 15q, 17p, and 18q losses. Adenomas with ≥2CAEs were considered molecular high-risk adenomas.

### Statistical Analysis

We determined (i) the prevalence of AAs and/or molecular high-risk adenomas at baseline in all included individuals, and (ii) calculated the OR for association between presence of either AA or molecular high-risk adenomas at baseline and development of me-CRC in the follow-up.

Two (controls) to one (case) incidence density matching was used to construct the nested case–control study, with matching based on sex and age range within ±5 years from the age of the index case. For control selection, the matched controls were required to be in the same risk set as the index case (27–29). That is, their follow-up duration had to be equal to or longer than that of the index case.

We performed univariate conditional logistic regression and estimated ORs with 95% confidence intervals (CI) for the association between the following baseline variables and me-CRC: age, adenoma status based on morphologic (AA or non-AA) and molecular (high-risk with ≥2 CAEs or low-risk with <2 CAEs) risk definitions (16). We also assessed other molecular high-risk definitions based on CNAs: (i) the presence of any type of ≥3 CNAs (gains and/or losses), or (ii) the presence of ≥3 gains, or (iii) the presence of ≥3 losses (30, 31).

To assess the association between molecular high-risk features present in the baseline adenoma and development of me-CRC adjusting for age at baseline and morphologic definition (AA or non-AA) of the adenoma, we also performed multivariate conditional logistic regression analyses. Sex was not included in the model because it has already been accounted for during the matching process. However, to account for differences in age within matched sets of case and controls, given that the matching was not based on exact same age, we included age at baseline in the model. We compared the predictive powers of fitted models using the Harrell's Concordance Statistic (32).

To explore whether the prognostic value of molecular features plays a role in only AA or only non-AAs, we evaluated possible interactions by including both definitions of adenoma (morphologic and molecular) as interaction terms in a conditional logistic regression model. Furthermore, we conducted a stratified time analysis to evaluate whether the presence of molecular high-risk features in the baseline adenoma is associated with the development of me-CRC in the short term. Finally, to check for possible differences in the timing of development of me-CRC between individuals who had AAs and those with non-AAs, as well as between those with high and low molecular risk, we performed a log-rank analysis. A Kaplan–Meier chart was incorporated to visualize the results. *P* values < 0.05 were considered statistically significant. All analyses were performed using R statistical software (version 3.6.2).

### Ethics Statement

#### Patient Consent for Publication

All living individuals included were provided with written information about the study and could opt out of the study. The study was conducted in accordance with the ethical principles for medical research of the declaration of Helsinki. The use of archival tissue and patient data were performed in compliance with the “Code for Proper Secondary Use of Human Tissue in the Netherlands” formulated by the Federation of Dutch Medical Scientific Societies.

#### Ethics Approval

This study involves human participants and was approved by the Regional Committee for Medical and Health Research Ethics of South-Eastern Norway (2014/2352) and by the Institutional Review Board for Human Research at the Netherlands Cancer Institute (IRBdm18-148).

### Data Availability Statement

The genomic data used in the current study are available in the European Genomes and phenomes Archive (ega-archive.org), with reference number EGAS00001007039. All other data in this article can be obtained from the corresponding author upon reasonable request.

## Results

### Prevalence of High-risk Molecular Features in AA and Non-AAs at Baseline Colonoscopy

**FIGURE 2:**
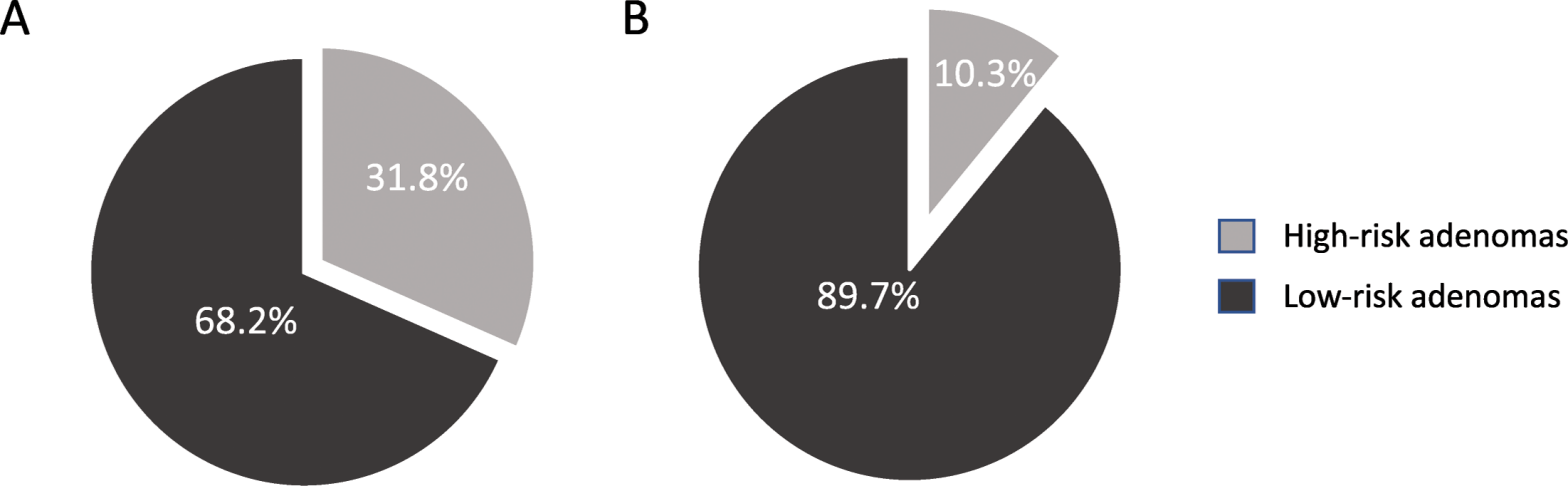
Proportion of molecular high-risk adenomas among the AAs (**A**) and non-AAs (**B**). Molecular high-risk adenomas are defined by the presence of ≥2 CAEs.

From the 744 selected individuals, we retrieved 634 FFPE adenomas, from which DNA of good quality was isolated from 594 samples (6.3% drop-out) and DNA copy-number status was successfully determined in 592 (0.3% drop-out). Of these, we were able to determine the morphologic adenoma classification in 529 individuals at baseline colonoscopy (Fig. 1A). In the 529 individuals with adenomas, 267 (50.5%) were AAs and 112 (21.2%) were molecular high-risk adenomas (adenomas with ≥2 CAEs). Of the latter, 85 (75.9%) were AAs and 27 (24.1%) were non-AAs (Table 1). From all AAs 31.8% were also molecular high-risk (85/267), and from all non-AAs 10.3% were also molecular high-risk (27/262; Table 1; Fig. 2).

**TABLE 1:** Distribution of the morphologic and molecular classifications of the adenomas included in the study

	AA (%)	nAA (%)	Total (%)
High	85 (31.8)	27 (10.3)	112 (21.2)
Low	182 (68.2)	235 (89.7)	417 (78.8)
Total	267 (50.5)	262 (49.5)	529 (100)

Abbreviations: AA, advanced adenoma; nAA, non-advanced adenoma; High, molecular high-risk adenoma defined by the presence of ≥2 CAEs; Low, molecular low-risk adenoma defined by 0–1 CAEs (16).

### Association Between Presence of an AA or a Molecular High-risk Adenoma at Baseline Colonoscopy and me-CRC

From the 529 adenomas, eight were excluded from further analysis because of lack of follow-up. From the remaining 521 adenomas, we were able to match 375 adenomas (125 cases and 250 controls), thus surpassing the sample size requirements (Fig. 1B). An overview of the characteristics of cases and controls is depicted in Table 2, showing the proportion of AAs and molecular high-risk adenomas in cases (individuals who developed me-CRC) and controls (individuals who did not).

**TABLE 2:** Characteristics of matched cases and controls of the adenoma cohort

	Cases (*n* = 125)	Controls (*n* = 250)	Total (*n* = 375)
Median follow-up, years, (IQR)	4.2 (1.5–8.0)	12.8 (7.9–16.3)	10.5 (4.5–14.7)
Sex, *n* (%)			
Male	55 (44)	110 (44)	165 (44)
Female	70 (56)	140 (56)	210 (56)
Median age, years, (IQR)	70 (61–77)	69 (60–75)	69 (60–76)
Adenoma status (Morphologic definition), *n* (%)			
Non-advanced	40 (32)	145 (58)	185 (49.3)
Advanced	85 (68)	105 (42)	190 (50.7)
Adenoma status (Molecular risk definition), *n* (%)			
CAE <2	90 (72)	202 (80.8)	292 (77.9)
CAE ≥2	35 (28)	48 (19.2)	83 (22.1)
CNA <3	64 (51.2)	152 (60.8)	216 (57.6)
CNA ≥3	61 (48.8)	98 (39.2)	159 (42.4)
Loss <3	96 (76.8)	221 (88.4)	317 (84.5)
Loss ≥3	29 (23.2)	29 (11.6)	58 (15.5)
Gain <3	81 (64.8)	184 (73.6)	265 (70.7)
Gain ≥3	44 (35.2)	66 (26.4)	110 (29.3)

Abbreviations: CAE, cancer-associated event; CNA, copy-number alteration; IQR, interquartile range.

The results of the univariate conditional regression analyses on the association of baseline features with me-CRC are reported in Table 3. Age at baseline was significantly associated with me-CRC (OR, 1.15; 95% CI, 1.05–1.26; *P* = 0.004). Also, presence of an AA, as compared with non-AA, was significantly associated with me-CRC, with an OR of 2.95 (95% CI, 1.84–4.71; *P* < 0.001; Table 3). When considering the molecular risk definition of adenomas, we found no statistically significant association between the development of me-CRC and removal at baseline of adenomas presenting with ≥2 CAEs versus adenomas with <2 CAEs (OR, 1.64; 95% CI, 0.99–2.71; *P* = 0.056). Similarly, no significance was found for the presence ≥3 CNAs (OR, 1.50; 95% CI, 0.96–2.34; *P* = 0.075) or the presence of ≥3 gains alone (OR, 1.52; 95% CI, 0.95–2.43; *P* = 0.080). However, when considering adenomas with ≥3 losses compared with adenomas with <3 losses, we observed a significant association with the development of me-CRC (OR, 2.31; 95% CI, 1.30–4.12; *P* = 0.004; Table 3). Moreover, we observed that, in general, losses were significantly more frequent in adenomas from individuals who developed me-CRC (cases) than in adenomas from individuals who did not (controls; *P* = 0.036; Supplementary Fig. S1).

**TABLE 3:** Univariate conditional logistic regression analysis to determine the association of the presence of AA, molecular high-risk adenoma (CAEs, any DNA CNAs, losses, or gains) with the development of a me-CRC

Variable	OR (95% CI)	*P*
Age	1.147 (1.046–1.259)	0.004
Adenoma status (Morphological definition)		
Non-advanced	1 (Reference)	
Advanced	2.946 (1.842–4.713)	<0.001
Adenoma status (Molecular risk definition)		
CAE <2	1 (Reference)	
CAE ≥2	1.638 (0.988–2.713)	0.056
CNA <3	1 (Reference)	
CNA ≥3	1.497 (0.960–2.335)	0.075
Loss <3	1 (Reference)	
Loss ≥3	2.314 (1.301–4.118)	0.004
Gain <3	1 (Reference)	
Gain ≥3	1.522 (0.952–2.434)	0.080

Abbreviations: CAE, cancer-associated event; CNA, copy-number alteration.

In the multivariate analysis, age at baseline (OR, 1.14; 95% CI, 1.03–1.26; *P* = 0.012), the presence of AA at baseline (OR, 2.46; 95% CI, 1.50–4.01; *P* < 0.001) and presence of ≥3 losses (OR, 1.90; 95% CI, 1.02–3.54; *P* = 0.043) remained statistically significant (Tables 4 and 5). The Harrell's Concordance Statistic of this three-variable model showed 69.8% (95% CI, 61.6–78.0) predictive power, as opposed to 69.4% (95% CI, 61.0–77.8) in a multivariate analysis with both age at baseline and the presence of AA at baseline.

**TABLE 4:** Multivariate conditional logistic regression analysis to determine the joint association of age, the presence of AA, molecular high-risk adenoma (CAEs, any DNA CNAs) with the development of a me-CRC

	Multivariate analysis^a^		Multivariate analysis^b^	
Variable	OR (95% CI)	*P*	OR (95% CI)	*P*
Age	1.131 (1.024–1.249)	0.015	1.125 (1.019–1.241)	0.019
Adenoma status (Morphologic definition)				
Non-advanced	1 (Reference)		1 (Reference)	
Advanced	2.595 (1.596–4.219)	<0.001	2.653 (1.622–4.341)	<0.001
Adenoma status (Molecular risk definition)				
CAE <2	1 (Reference)		—	
CAE ≥2	1.362 (0.793–2.339)	0.263	—	—
CNA <3	—		1 (Reference)	
CNA ≥3	—	—	1.156 (0.712–1.874)	0.558

Abbreviations: CAE, cancer-associated event; CNA, copy-number alteration.

^a^Model includes age, morphologic definition of adenoma, molecular risk definition of adenoma (CAE <2 and CAE ≥2).

^b^Model includes age, morphologic definition of adenoma, molecular risk definition of adenoma (CNA <3 and CNA ≥3).

**TABLE 5:** Multivariate conditional logistic regression analysis to determine the association of age, the presence of AA, molecular high-risk adenoma (losses or gains) with the development of a me-CRC

	Multivariate analysis^a^		Multivariate analysis^b^	
Variable	OR (95% CI)	*P* value	OR (95% CI)	*P* value
Age	1.138 (1.029–1.258)	0.012	1.124 (1.019–1.240)	0.019
Adenoma status (Morphologic definition)				
Non-advanced	1 (Reference)		1 (Reference)	
Advanced	2.456 (1.504–4.010)	<0.001	2.656 (1.638–4.306)	<0.001
Adenoma status (Molecular risk definition)				
Loss <3	1 (Reference)		—	—
Loss ≥3	1.899 (1.020–3.538)	0.043	—	
Gain <3	—		1 (Reference)	
Gain ≥3	—	—	1.230 (0.748–2.023)	0.415

^a^Model includes age, morphologic definition of adenoma, molecular risk definition of adenoma (Loss <3 and Loss ≥3).

^b^Model includes age, morphologic definition of adenoma, molecular risk definition of adenoma (Gain <3 and Gain ≥3).

To further study the potential added value of presence of molecular features in addition to AA in the risk association with me-CRC, we also conducted an exploratory analysis in which we included an interaction term in the model representing the simultaneous presence of advanced morphologic features and molecular features (in the latter, considering CAEs or losses alone). Adding an interaction term did not improve the predictive ability of models with either CAEs or losses, as the interaction terms were not statistically significant (Table 6).

**TABLE 6:** Comparisons of subgroups to explore the interaction between morphologic and molecular risk definitions in the association with me-CRC risk

Comparison	OR (95% CI)	*P*	Comparison	OR (95% CI)	*P*
Non-advanced* CAE <2	1	—	Non-advanced* Loss <3		—
vs.			vs.		
Non-advanced* CAE ≥2	1.442 (0.471–4.419)	0.521	Non-advanced* Loss ≥3	1.992 (0.463–8.572)	0.355
Advanced* CAE <2	1	—	Advanced* Loss <3	1	—
vs.			vs.		
Advanced* CAE ≥2	1.338 (0.718–2.492)	0.359	Advanced* Loss ≥3	1.880 (0.952–3.715)	0.069
Advanced* CAE <2	1	—	Advanced* Loss <3	1	—
vs.			vs.		
Non-advanced* CAE ≥2	0.548 (0.184–1.630)	0.280	Non-advanced* Loss ≥3	0.807 (0.188–3.459)	0.772

Abbreviation: CAE, cancer-associated event.

Within the group of individuals with AA, there was no statistically significant difference in the odds of developing me-CRC between those classified as having high molecular risk and those with low molecular risk, both considering the presence of CAEs (OR, 1.34; 95% CI, 0.72–2.49; *P* = 0.359) or only losses (OR 1.88; 95% CI, 0.95–3.72; *P* = 0.069) as molecular risk. Similar results follow for individuals with non-AAs with either high or low molecular risk features as well as for individuals with non-AAs with high molecular risk in comparison to individuals with AAs with low molecular risk (Table 6).

Results of the stratified time analysis (Table 7) show that the presence of molecular high-risk features in the baseline adenoma was associated with the development of me-CRC within the first year after removal of the adenoma and not afterward. This was observed both for presence of CAEs (OR, 3.84; 95% CI, 1.16–12.70; *P* = 0.028) and for losses only (OR, 8.29; 95% CI, 1.78–38.69; *P* = 0.007). Considering the morphologically defined AA, a significant association with me-CRC was observed within the first year (OR, 16.36; 95% CI, 2.10–127.70; *P* = 0.008) and after 3 years (OR, 2.56; 95% CI, 1.45–4.53; *P* = 0.001) of adenoma removal.

**TABLE 7:** Stratified time analysis showing the effect of molecular risk definitions (CAEs or losses) and the morphologic risk definition of adenoma on developing me-CRC

			CAE		Loss		Advanced adenoma	
Follow-up time	Cases (*n* = 125)	Controls (*n* = 250)	OR (95% CI)^a^	*P*	OR (95% CI)^b^	*P*	OR (95% CI)^c^	*P*
≤ 1 year	20	40	3.836 (1.159–12.700)	0.028	8.293 (1.777–38.69)	0.007	16.36 (2.096–127.700)	0.008
>1–≤2 years	19	38	2.862 (0.814–10.050)	0.101	2.195 (0.576–8.362)	0.249	2.189 (0.5776–8.297)	0.249
>2–≤3 years	14	28	2.171 (0.364–12.940)	0.395	1.443 (0.187–11.120)	0.725	1.897 (0.4585–7.847)	0.377
>3 years	72	144	0.958 (0.473–1.940)	0.905	1.583 (0.710–3.530)	0.262	2.559 (1.446–4.529)	0.001

Abbreviation: CAE, cancer-associated event.

^a^The estimates of the OR correspond to the effect of molecular high-risk adenoma defined by CAE ≥2 with molecular low-risk adenoma (i.e., CAE <2) as the reference category.

^b^The estimates of the OR correspond to the effect of molecular high-risk adenoma defined by having Loss ≥3, with Loss <3 as the reference category.

^c^The estimates of the OR correspond to the effect of advanced adenoma, with non-advanced adenoma as the reference category.

The Kaplan–Meier analyses, done to evaluate whether molecular features would lead to a shorter time to development of me-CRC, are shown in Supplementary Fig. S2. These results show that for both molecular risk definitions considered (CAEs or losses alone), individuals with AAs with molecular high-risk features have a shorter time to development of me-CRC in the first 5 years than individuals with AAs with molecular low-risk features, though this was not statistically significant (Supplementary Fig. S2A and S2B). The number of individuals with non-AAs harboring molecular high-risk features was too low to draw any conclusions (Supplementary Fig. S2C and S2D). Considering the morphologically defined features alone, no differences in time to development of me-CRC were observed between AA versus non-AA (Supplementary Fig. S2E).

## Discussion

In the current study, we hypothesized that when an individual carries an adenoma already showing molecular alterations highly associated with progression to cancer (i.e., CAEs), such an individual may be prone to later develop another adenoma that also accumulates these CAEs and does progress into a metachronous cancer (me-CRC). Here, we found that a molecular classification of baseline adenomas, based on DNA CNAs, in particular DNA copy-number losses, is associated with the development of me-CRC. Yet, the currently used morphologic determination of AA showed a stronger association with me-CRC.

Our results show that only a minority of AAs (32%) harbor molecular high-risk features, namely CAEs, in line with previously published work (19). Furthermore, we observed that having a molecular high-risk adenoma (based on copy-number losses) removed at baseline colonoscopy was associated with a 2.3 times increased odds of me-CRC. Having an AA was associated with a 2.9 times increased odds of developing me-CRC, similarly to previous publications (33–35). When considering the independent prognostic value of the two classifications (AA and molecular high-risk adenoma), as assessed in a multivariate analysis, having an AA removed was a stronger predictor of risk of me-CRC (OR 2.5) than having molecular high-risk adenoma removed (OR 1.9), although the latter (copy-number losses) was still an independent prognostic factor. Also, in our exploratory stratified time analysis, the presence of molecular high-risk features (CAEs or copy-number losses only) showed a significant association with the risk of developing me-CRC within the first year after adenoma removal, but this association was less strong than for the AA classification.

In the Kaplan–Meier analysis of time to develop me-CRC, individuals with AAs harboring molecular high-risk features (CAEs or copy-number losses) tended to develop me-CRC earlier than those with low-risk features (*P* = 0.07). This suggests a potential additional value of molecular features next to using the AA definition. However, when exploring the interactions between AAs and molecular features, in particular the risk in AA with versus without ≥3 copy-number losses, the observed OR of 1.88 was not significant (*P* = 0.069).

Chromosomal instability, leading to gains and losses of whole (or big parts of) chromosomes, is well established as one of the crucial steps toward malignancy (15, 20, 36). Previously we have shown that specific gains and losses (CAEs) are associated with the transition from a benign lesion (adenoma) to cancer (16, 17). Therefore, CAEs are good predictors of risk for a given lesion to progress to cancer. However, lesions detected during colonoscopy are removed, and the natural history is interrupted. Therefore, it is not unlikely that alterations detected in a removed adenoma are not only part of the natural history of that particular adenoma, but also associated to an individual's risk to develop similar adenomas and colorectal cancers later on. Thus, all adenomas within an individual may share features that relate to future colorectal cancer risk. Similar associations are assumed for morphologic features size, dysplasia, and (until recently) villous histology, which are the basis of the present surveillance guidelines.

In this study, we show that although molecular high-risk features in baseline lesions, particularly DNA copy-number losses, are associated with an increased risk (OR, 1.9) for me-CRC, this association is not as strong as for the presence of an AA at baseline (OR, 2.5).

A recent small study, analyzing DNA CNAs in adenomas detected at baseline colonoscopy both in individuals that later developed cancer (*n* = 12) and individuals that did not develop cancer (*n* = 37), showed a higher burden of DNA CNAs in adenomas from individuals who developed me-CRC compared with adenomas from individuals who did not develop colorectal cancer (37). These results are also in line with our findings, both when looking at the specific CAEs and at the total burden of DNA copy-number losses (Supplementary Fig. S1). However, in that published small series, the morphologic features of the analyzed adenomas were not considered, therefore the predictive value of a molecular risk classification could not be compared with the classification into advanced and non-AA. To the best of our knowledge, our study is the first study that directly compares the association between the risk of me-CRC and presence of molecular features (CNAs) to the association between risk of me-CRC and presence of AAs at baseline colonoscopy.

Even if a risk classification based on morphologic features of adenomas is better than based on molecular features, the current high- and low-risk adenoma morphologic classification is not accurate enough to predict risk of colorectal cancer and probably leads to excessive colonoscopy surveillance (38). However, our results show that AA is a better risk factor than DNA CNAs for me-CRC. Apparently, the presence of an AA is capturing some hidden biology impacting future colorectal cancer risk, other than the amount of DNA CNAs in the baseline lesion. It is known that CNAs are late events in the progression of certain lesions and are considered the final trigger to become cancer (36). Therefore, it is possible that other types of molecular features in adenomas, for example mutations, which are earlier events in the development of cancer, could aid in the improvement on the prediction of risk to develop new lesions and cancer.

There are several strong points in this study. We performed a study with a nested case–control study of a national, longitudinal surveillance cohort. Therefore, we have complete registration of an individual's me-CRC diagnosis over time. Data were collected prospectively and using a nested case–control design allowed efficient collection of additional clinical data in chart reviews. Such a large series (*n* = 529) allowed us to have sufficient power to compare matched cases (me-CRC) and controls (no me-CRC) with similar follow-up time. In addition, through this design, we were able to use the clinically most relevant endpoint me-CRC rather than intermediate endpoints such as AAs used in many previous studies have used (39–41). Furthermore, we had detailed clinical data of use of colonoscopy, treatment, and follow-up. In particular, the detailed information on morphologic features of the adenomas, made it possible to compare this with the molecular features of the adenomas.

There are also some limitations in our study that should be addressed. First, we did not consider the number of polyps as covariable, which is one of the clinical features used in clinical practice to assess risk of me-CRC. However, we consciously included only individuals with single polyps at baseline to limit the complexity of the study. Second, case–control studies are more prone to biases than prospective studies. However, as colorectal cancer can be considered a relatively rare event, a prospective cohort study to determine the relative risk of developing colorectal cancer is not feasible. Third, clinical practice with regards to surveillance recommendations, and removal techniques have developed over time, which may affect the risk of me-CRC. However, as all included individuals in this study were subjected to the same clinical practice, this would not affect the relative comparisons. Furthermore, because the natural history of the development of colorectal cancer takes years, all studies with a sufficiently long follow-up will be facing change of clinical practice over time.

In summary, we have shown that DNA copy-number changes, in particular DNA copy-number losses, were indeed associated with an individual's risk of developing me-CRC. Yet, the presence of a molecularly-defined high-risk adenoma did not better predict the risk for me-CRC than the presence of a morphologically defined AA. To the best of our knowledge, this is the first study that directly compares the presence of molecular features (DNA CNAs) with presence of AAs, at baseline colonoscopy and its association with risk of me-CRC.

## Supplementary Material

Supplementary Figure 1 Supplementary Figure 1. Comparison of losses between cases and controls.

Supplementary Figure 2 Supplementary Figure 2 shows a Kaplan-Meier analysis evaluating the timing of development of me-CRC in advanced adenomas with or without molecular high-risk features.
