# Supplementary material for: Risk Prediction of Metachronous Colorectal Cancer from Molecular Features of Adenomas: A Nested Case–Control Study
Source: Cancer Res Commun. 2023 Nov 13;3(11):2292–301. doi: 10.1158/2767-9764.CRC-23-0186 (PMC10642372; doi:10.1158/2767-9764.CRC-23-0186)
Supplement: Supplementary Figure 1 — Comparison of losses between cases and controls. [file crc-23-0186-s01.pdf]

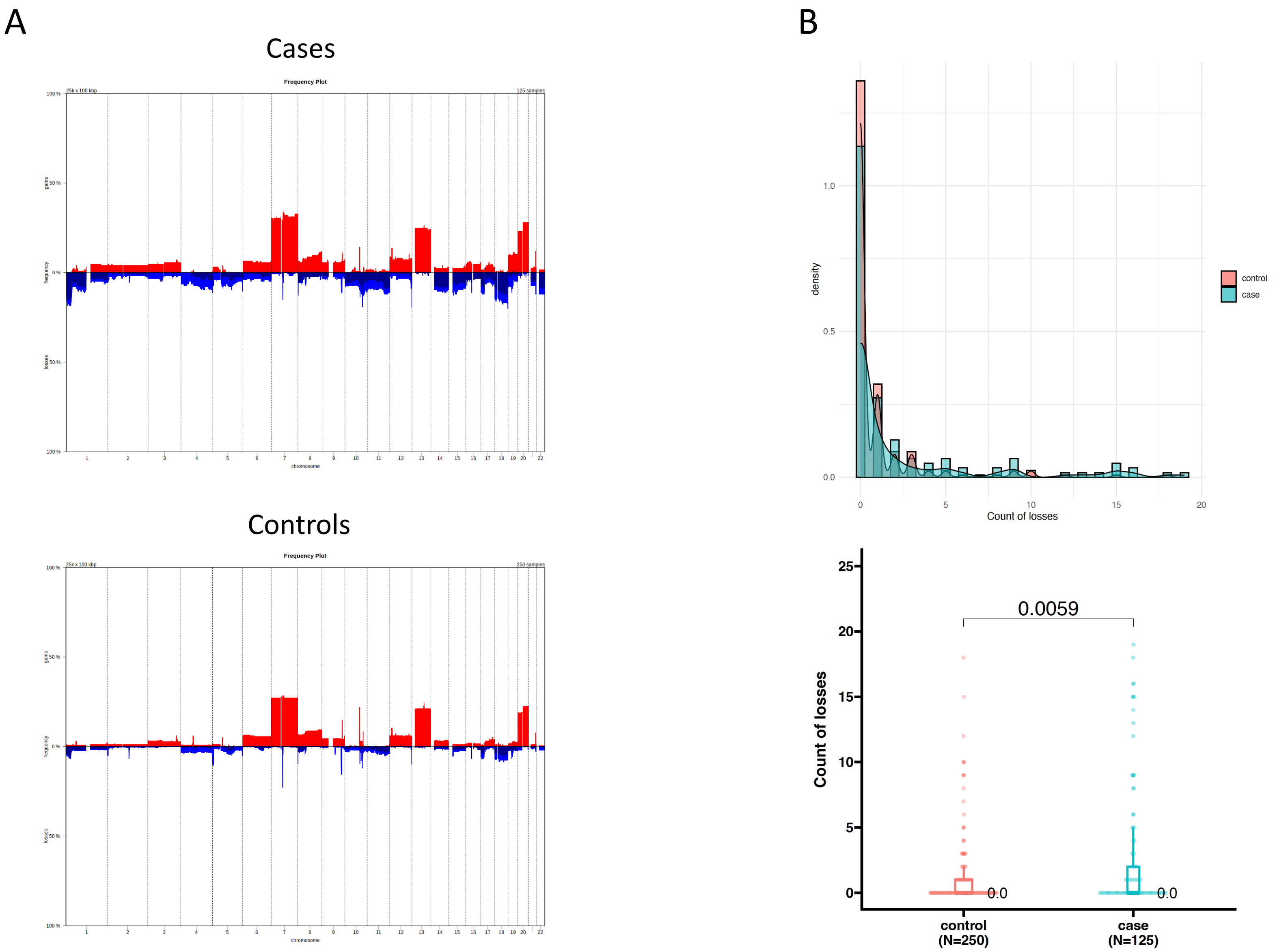

Supplementary Figure 1. Comparison of losses between cases and controls. A, Frequency plot of DNA copy number alterations in adenomas from individuals that were diagnosed with a metachronous CRC (cases) and from individuals without metachronous CRC with the same follow-up (controls); gains (red) and losses (blue). B, distribution and count of losses in cases (green) and controls (zalm).
