# Supplementary material for: Risk Prediction of Metachronous Colorectal Cancer from Molecular Features of Adenomas: A Nested Case–Control Study
Source: Cancer Res Commun. 2023 Nov 13;3(11):2292–301. doi: 10.1158/2767-9764.CRC-23-0186 (PMC10642372; doi:10.1158/2767-9764.CRC-23-0186)
Supplement: Supplementary Figure 2 — shows a Kaplan-Meier analysis evaluating the timing of development of me-CRC in advanced adenomas with or without molecular high-risk features. [file crc-23-0186-s02.pdf]

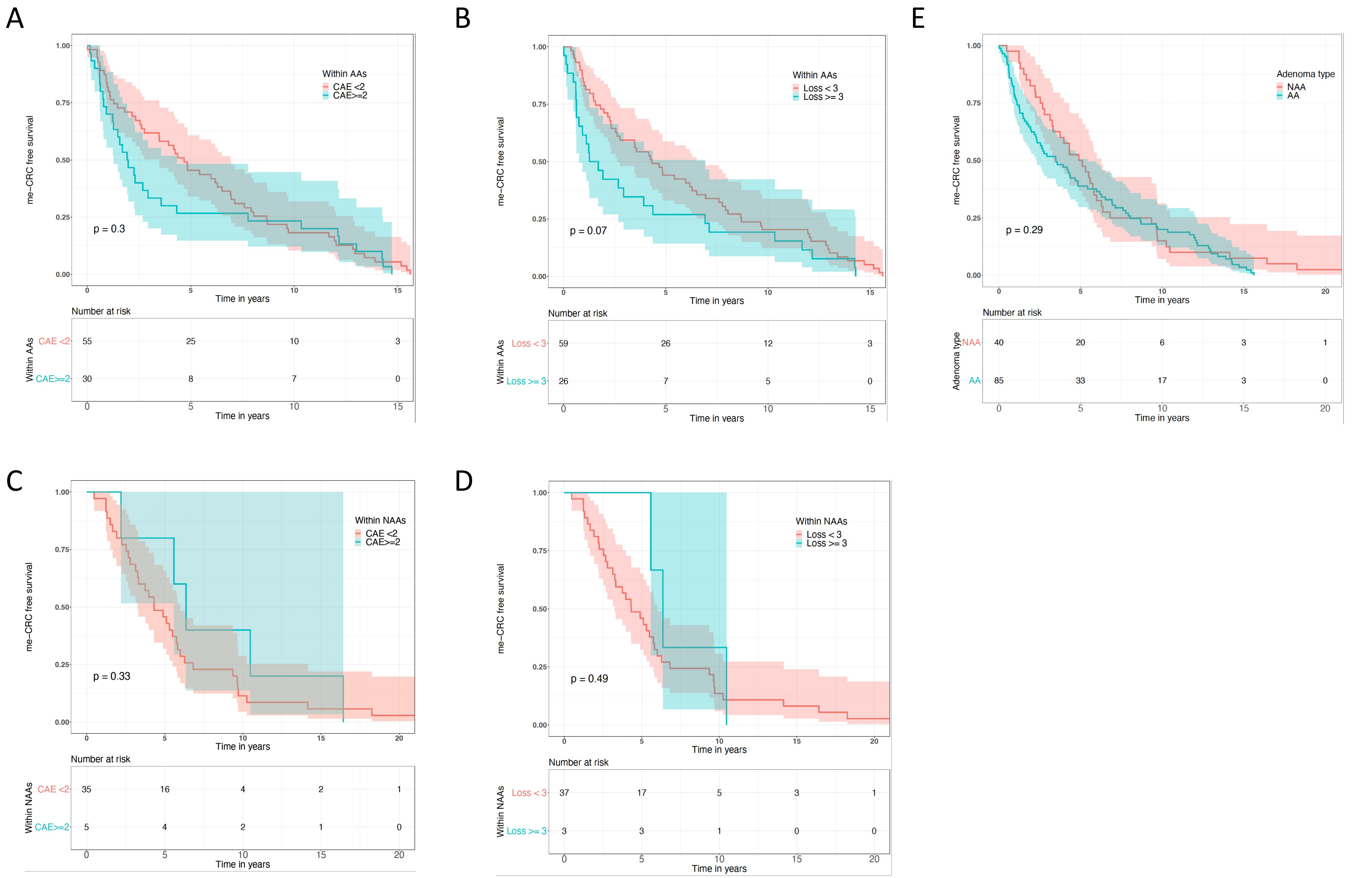

Supplementary Figure 2. Kaplan-Meier analysis evaluating the timing of development of me-CRC in advanced adenomas (**A**, **B**) with or without molecular high-risk features, namely CAEs (**A**) or losses alone (**B**), or non-advanced adenomas (**C**, **D**) with or without molecular high-risk features, namely CAEs (**C**) or losses alone (**D**), or in advanced adenomas compared to non-advanced adenomas (**E**).
